# CRISPR/dCas9-Mediated Gene Silencing in Two Plant Fungal Pathogens

**DOI:** 10.1128/msphere.00594-22

**Published:** 2023-01-19

**Authors:** Yun-mu Zhang, Lu Zheng, Kabin Xie

**Affiliations:** a National Key Laboratory of Crop Genetic Improvement, Hubei Hongshan Laboratory, Huazhong Agricultural University, Wuhan, Hubei Province, People’s Republic of China; b Jingchu University of Technology, Jingmen, Hubei Province, People’s Republic of China; c Hubei Key Laboratory of Plant Pathology, Huazhong Agricultural University, Wuhan, Hubei Province, People’s Republic of China; University of Georgia

**Keywords:** CRISPR interference, gene silencing, dCas9, *Magnaporthe oryzae*, *Ustilaginoidea virens*, functional genomics, CRISPR/Cas, filamentous fungi

## Abstract

Magnaporthe oryzae and Ustilaginoidea virens are two filamentous fungal pathogens that threaten rice production worldwide. Genetic tools that permit fast gene deletion and silencing are of great interest for functional genomics of fungal pathogens. As a revolutionary genome editing tool, clustered regularly interspaced palindromic repeats (CRISPR) and CRISPR-associated protein 9 (Cas9) enable many innovative applications. Here, we developed a CRISPR interference (CRISPRi) toolkit using nuclease activity dead Cas9 (dCas9) to silence genes of interest in M. oryzae and *U. virens*. We optimized the components of CRISPRi vectors, including transcriptional repression domains, dCas9 promoters, and guide RNA (gRNA) promoters. The CRISPRi tool was tested using nine gRNAs to target the promoters of *MoATG3*, *MoATG7*, and *UvPal1*. The results indicated that a single gRNA could direct the dCas9-fused transcriptional repression domain to efficiently silence the target gene in M. oryzae and *U. virens*. In both fungi, the target genes were repressed >100-fold, and desired phenotypes were observed in CRISPRi strains. Importantly, we showed that multiple genes could be easily silenced using polycistronic tRNA-gRNA in CRISPRi. Furthermore, gRNAs that bind different promoter regions displayed variable repression levels of target genes, highlighting the importance of gRNA design for CRISPRi efficiency. Together, this study provides an efficient and robust CRISPRi tool for targeted gene silencing in M. oryzae and *U. virens*. Owing to its simplicity and multiplexity, CRISPRi will be a useful tool for gene function discovery in fungal pathogens.

**IMPORTANCE** Many devastating plant diseases are caused by fungal pathogens that evolve rapidly to adapt to host resistance and environmental changes. Therefore, genetic tools that enable fast gene function discovery are needed to study the pathogenicity and stress adaptation of fungal pathogens. In this study, we adopted the CRISPR/Cas9 system to silence genes in Magnaporthe oryzae and Ustilaginoidea virens, which are two dominant fungal pathogens that threaten rice production worldwide. We present a versatile and robust CRISPRi toolkit that represses target gene expression >100-fold using a single gRNA. We also demonstrated that CRISPRi could simultaneously silence multiple genes using the tRNA-gRNA strategy. The CRISPRi technologies described in this study would accelerate the functional genomics of fungal pathogens.

## INTRODUCTION

Fungal pathogens, which can cause approximately 70 to 80% of total plant diseases (1), are among the dominant causal agents of plant diseases that threaten food safety across the world (2, 3). Rice blast fungus (Magnaporthe oryzae) is one of the most important pathogens and causes approximately 30% of rice production losses in the world (3–5). Recently, Ustilaginoidea virens, which infects rice flowers and causes rice false smut, has emerged as a new threat to rice yield and grain quality (6, 7). Sophisticated gene knockout tools, including gene deletion using homologous recombination (HR) and random mutagenesis using T-DNA insertion, are widely used to generate mutants of M. oryzae (8, 9) and *U. virens* (10, 11). T-DNA insertion and HR-mediated gene deletion are laborious for large-scale studies. In particular, genetic tools that can simultaneously knock out or knock down multiple genes are lacking in fungal pathogens. Hence, new technologies for gene knockout and silencing are of great interest for the functional genomics of M. oryzae and *U. virens*.

**FIG 1:**
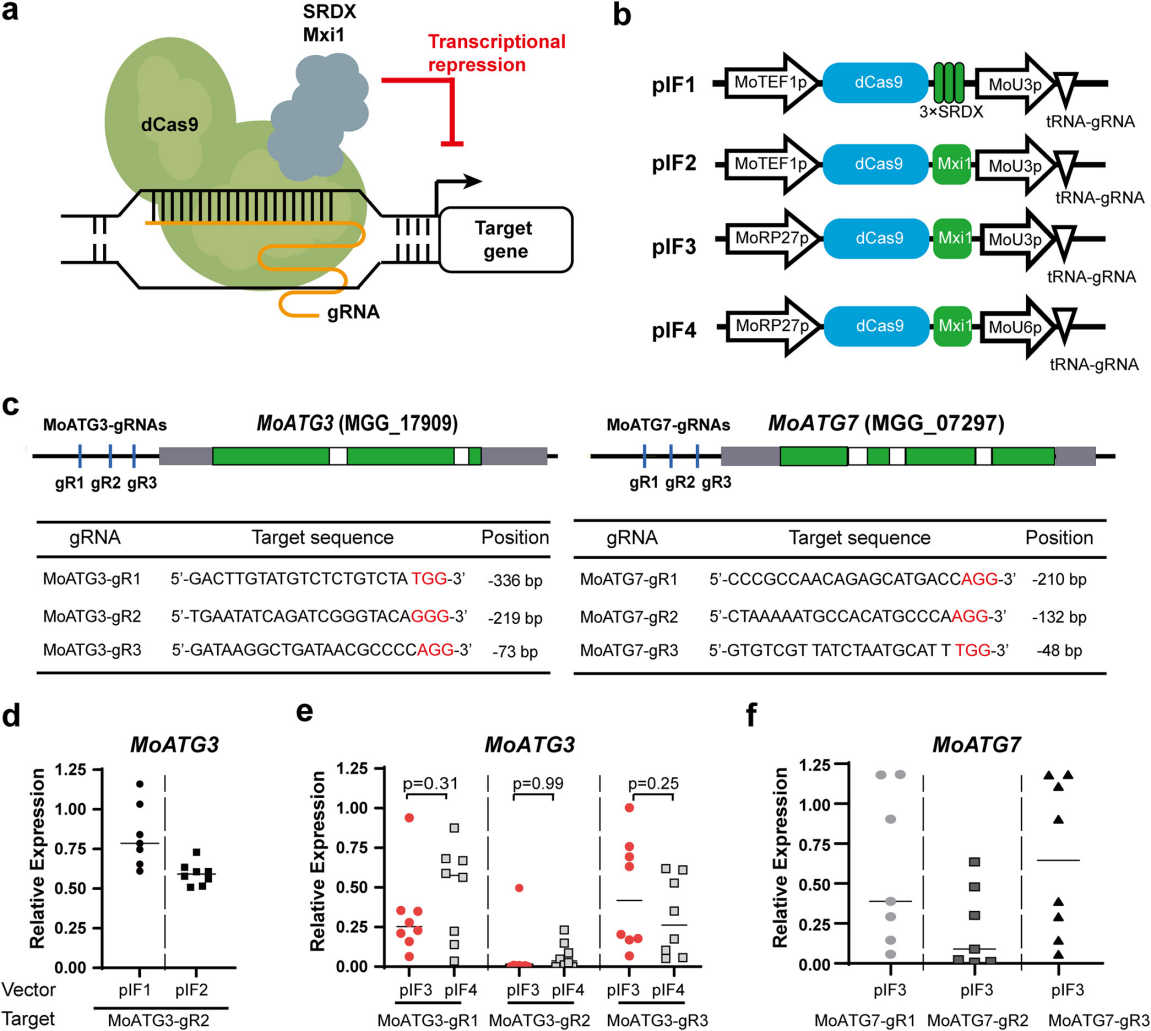
Targeted gene silencing in M. oryzae. (a) Schematics of targeted gene silencing using the dCas9-fused transcriptional repressor. (b) Structure of CRISPRi vectors developed in this study. MoTEF1p, M. oryzae *TEF1* promoter; MoRP27p, M. oryzae *RP27* promoter; *MoU3p* and *MoU6p*, M. oryzae U3 and U6 snoRNA promoters. The single gRNA was expressed as a tRNA-gRNA fusion. See Fig. S2 and S3 for gRNA cloning site sequences. (c) Positions and sequences of the targeting sites of *MoATG3* and *MoATG7*. The upper panel shows the position of gRNAs (vertical lines), introns (white box), untranslated regions (gray box), and protein coding regions (green boxes) of target genes. The gRNA information is shown under the plots. Red letters indicate PAM sequences. Positions indicate the distance between PAM and transcription start sites. (d to f) Relative expression of *MoATG3* and *MoATG7* in CRISPRi strains. The empty vector transformants were used as a reference to calculate the relative expression level of target genes. Each point indicates the relative expression of an individual transformant (*n* = 3 technical replicates). The lines indicate the mean relative expression of all transformants. *P* values of Student *t* test (*n* = 8) are shown in panel e.

In the past 10 years, CRISPR/Cas-mediated genome editing has revolutionized the life sciences (12). Among the different CRISPR/Cas systems, CRISPR/Cas9 from Streptococcus pyogenes is the first and most widely used CRISPR system for genome editing. Cas9 nuclease is directed by a single guide RNA (gRNA) to cleave the target DNA, which matches the 20-nucleotide (nt) guide sequence of gRNA and contains a protospacer adjacent motif (PAM; 5′-NGG-3′ for Streptococcus pyogenes Cas9). During the repair of the double-stranded DNA break (DSB) introduced by Cas9 cleavage, error-prone nonhomologous end joining (NHEJ) repair can introduce small indels to disrupt protein translation. On the other hand, the homology-directed repair (HDR) of DSB could be engineered for precise gene deletion and replacement. In addition to genome editing, the nuclease activity dead Cas9 (dCas9), which contains D10A and H840A mutations, is engineered to activate/suppress the transcription of target genes (13–15) (Fig. 1a). Furthermore, CRISPR/Cas9-mediated base editing and prime editing permit precise modifications of gene sequences (16–18). Owing to its simplicity, large-scale genetic screening could be implemented using pooled and arrayed gRNA libraries in animals, plants, and bacteria (19–22). These various CRISPR technologies have revolutionized basic and translational research in agriculture.

10.1128/msphere.00594-22.2 FIG S2 Sequence of the *MoU3* promoter and gRNA cloning site. Yellow indicates two BsaI sites (GGTCTCN, underlined) to insert the tRNA-gRNA fragment into the pIF1, pIF2 and pIF3 vectors. Download FIG S2, JPG file, 0.8 MB. Copyright © 2023 Zhang et al. 2023 Zhang et al. https://creativecommons.org/licenses/by/4.0/ This content is distributed under the terms of the Creative Commons Attribution 4.0 International license.

10.1128/msphere.00594-22.3 FIG S3 Sequence of the *MoU6* promoter and gRNA cloning site. Yellow indicates two BsaI sites (GGTCTCN, underlined) to insert the tRNA-gRNA fragment into the pIF4 vector. Download FIG S3, JPG file, 1.0 MB. Copyright © 2023 Zhang et al. 2023 Zhang et al. https://creativecommons.org/licenses/by/4.0/ This content is distributed under the terms of the Creative Commons Attribution 4.0 International license.

Classic CRISPR/Cas9 is also widely used for genome editing in more than 30 fungal species, including a few fungal pathogens (23, 24). However, Cas9 is cytotoxic to some fungal species, including M. oryzae (25). Therefore, targeted gene knockout via NHEJ repair is not feasible in many fungal species. Instead, transient expression of Cas9/gRNA was used to increase the efficiencies of HR-mediated gene deletion in M. oryzae (25, 26), *U. virens* (27), and other fungi (28). The dCas9-mediated transcriptional regulation was used in Saccharomyces cerevisiae (13), Yarrowia lipolytica (29), and the fungal pathogen Candida albicans (30, 31), which provides a powerful approach for gene function discovery and metabolic process reprogramming (32).

In this study, we adopted CRISPR/Cas9 technology and developed a CRISPR interference (CRISPRi) toolkit for targeted gene silencing in M. oryzae and *U. virens* (Fig. 1a). We systematically optimized the components of CRISPRi vectors and achieved more than 100-fold repression of target genes in M. oryzae and *U. virens*. More importantly, two or more genes could be simultaneously silenced with high efficiencies using the polycistronic tRNA-gRNA strategy. This study expands the CRISPR technologies in filamentous fungi and provides a powerful gene silencing toolkit for functional genomics of fungal pathogens.

## RESULTS

### Optimization of the transcriptional repression domains of CRISPRi in *M. oryzae*.

To test dCas9-mediated gene silencing in M. oryzae, we constructed a binary vector pIF1 based on the pCAMBIA1300 backbone (Fig. 1a and b). This vector fused dCas9 with triple repeats of SUPERMAN Repression Domain X (3×SRDX; LDLDLELRLGFA, see Fig. S1 in the supplemental material for the DNA sequence), which displayed effective targeted gene repression in plants (33, 34). The U3 small nucleolar RNA promoter (*MoU3p*; see Fig. S2 for the promoter sequence) from M. oryzae was used to express gRNA, and the translation elongation factor 1 (*MoTEF1*, MGG_03641) promoter was used to express dCas9-SRDX. Of note, all single gRNAs were expressed as tRNA-gRNA fusions in this study since tRNA could efficiently boost gRNA expression and is precisely processed by endogenous RNase (35). An Aspergillus nidulans *trpC* (*AnTrpC*) promoter is used to express the hygromycin B phosphotransferase (*HPT*) for selection of positive transformants in *Agrobacterium*-mediated transformations (ATMT). To test the efficiencies of pIF1, we designed a gRNA (MoATG3-gR2) to specifically target the promoter of M. oryzae autophagy-related gene 3 (*MoATG3*, MGG_02959). *MoATG3* encodes a ubiquitin-conjugating enzyme (E2) that regulates autophagy and is required for the pathogenicity of M. oryzae (36, 37). The targeting site of MoATG3-gR2 is located 219 bp upstream of the transcription start site (TSS) of *MoATG3* (Fig. 1c). We transformed the pIF1-MoATG3-gR2 plasmid into M. oryzae through ATMT and determined target gene expression by reverse transcription-quantitative PCR (RT-qPCR). The colonies transformed with the empty vector pIF1 displayed normal growth, suggesting that dCas9 is not cytotoxic in M. oryzae. In eight randomly selected pIF1-MoATG3-gR2 colonies, the relative expression of *MoATG3* in five transformants was reduced 1.2- to 1.6-fold compared to the empty vector control (CK; Fig. 1d), suggesting that dCas9-SDRX is amenable to repress target genes in M. oryzae.

10.1128/msphere.00594-22.1 FIG S1 Sequence of codon-optimized 3×SRDX. Download FIG S1, JPG file, 0.3 MB. Copyright © 2023 Zhang et al. 2023 Zhang et al. https://creativecommons.org/licenses/by/4.0/ This content is distributed under the terms of the Creative Commons Attribution 4.0 International license.

To test the CRISPRi efficiencies with different transcriptional repressors, the pIF2 vector was constructed by combining dCas9 with the Mxi1 repressor domain (Fig. 1b). Mxi1 has been validated as a robust transcriptional repressor for CRISPRi in yeast (13). In eight transformants expressing dCas9-Mxi1 and MoATG-gR2, *MoATG3* mRNA was reduced 1.4- to 2-fold compared to the CK strains (Fig. 1d). Although dCas9-Mxi1 displayed slightly higher transcriptional repression activity than dCas9-3×SDRX, none of them suppressed the target gene >2-fold. These data demonstrate the feasibility of CRISPRi in M. oryzae; however, further optimization is required to increase its efficiency.

### Enhancing CRISPRi efficiency by optimizing the promoters of dCas9.

We next sought to enhance CRISPRi efficiency by optimizing the promoters of dCas9 and gRNA. To this end, we constructed pIF3, which uses the M. oryzae ribosomal protein 27 (*MoRP27*, MGG_10827) promoter to express dCas9-Mxi1. Because the size of T-DNA affects the efficiency of *Agrobacterium*-mediated transformation (38), we further compacted the components in T-DNA. To this end, herpes simplex virus thymidine kinase (*HSVtk*) and *HPT* selection markers were fused using T2A peptide and expressed with the *Cochliobolus heterostrophus* glyceraldehyde-3-phosphate dehydrogenase (*ChGPD*) promoter. The CRISPRi efficiency of pIF3 was also tested with MoATG3-gR2. We analyzed the expression of target genes in eight positive colonies. The results showed that *MoATG3* was silenced in all transformants, including five transformants with >100-fold repression of the target gene (Fig. 1e). The pIF3 vector was further validated using MoATG3-gR1 and MoATG3-gR3. The targeting sites of these two gRNAs are located 336 and 73 bp upstream of the TSS of *MoATG3* (Fig. 1c). MoATG3-gR1 and MoATG1-gR3 repressed target genes up to 27- and 19-fold, respectively (Fig. 1e). We also observed that MoATG3-gR1 and MoATG3-gR3 were less effective in some transformants, including 12.5% (1/8) of MoATG3-gR1 transformants and 50% (4/8) of MoATG3-gR3 transformants with <2-fold repression of target genes. These data implied that different gRNAs had variable robustness in CRISPRi. Together, these data demonstrate that optimizing dCas9 expression with the *MoRP27* promoter could drastically enhance CRISPRi efficiencies in M. oryzae.

The promoter expressing gRNA is important for CRISPR/Cas9 gene editing efficiencies. In fungi, many studies have used the U6 snoRNA promoter to express gRNA for CRISPR/Cas9 (28). We therefore exploited the efficiencies of CRISPRi vectors using U6 promoters. To this end, we designed the pIF4 vector, which expresses gRNA with the U6 snoRNA promoter of M. oryzae (*MoU6p*; see Fig. S3 for sequence). Using the same three gRNAs targeting the *MoATG3* promoter, the pIF4 vector displayed comparable CRISPRi efficiencies as the pIF3 vector (Fig. 1b and e). Of note, we fused gRNA with a tRNA in these experiments, and the tRNA sequence also functions as a potential intergene promoter to boost gRNA expression (35). We concluded that both the U3 and U6 promoters are highly efficient in expressing tRNA-gRNA for CRISRPi in M. oryzae.

To further validate CRISPRi using pIF3, three gRNAs were designed to target the promoter of M. oryzae autophagy-related gene 7 (*MoATG7*; Fig. 1c and f), which encodes a ubiquitin-activating enzyme (E1) and cooperates with *MoATG3* to regulate autophagosome formation. The targeting sites of these three gRNAs were located 210, 132, and 48 bp upstream of the TSS of *MoATG7* (Fig. 1c). We analyzed target gene expression in 8 randomly selected transformants for each gRNA. *MoATG7* was reduced >2-fold in approximately 50% of transformants. The highest repression (20-fold) was obtained in transformants expressing MoATG7-gR2, whose targeting site was located 132 bp upstream of the TSS. This is consistent with previous reports in animal and yeast cells (13) that the distance between the targeting site and TSS is critical for CRISPRi efficiencies. However, among the two tested target genes, the gRNAs with the highest CRISPRi efficiencies were located at different positions, implying that the optimal target site is also dependent on the chromatin accessibility of the target site (39) and the 20-nt guide sequence of the gRNA (40). In these experiments, all six gRNAs efficiently repressed the expression of target genes using CRISPRi, implying the high robustness of target gene silencing using *MoRP27* promoter expressed dCas9-Mxi1 in pIF3 and pIF4 vectors in M. oryzae.

### *MoATG3* CRISPRi strains lost pathogenicity to infect rice leaves.

**FIG 2:**
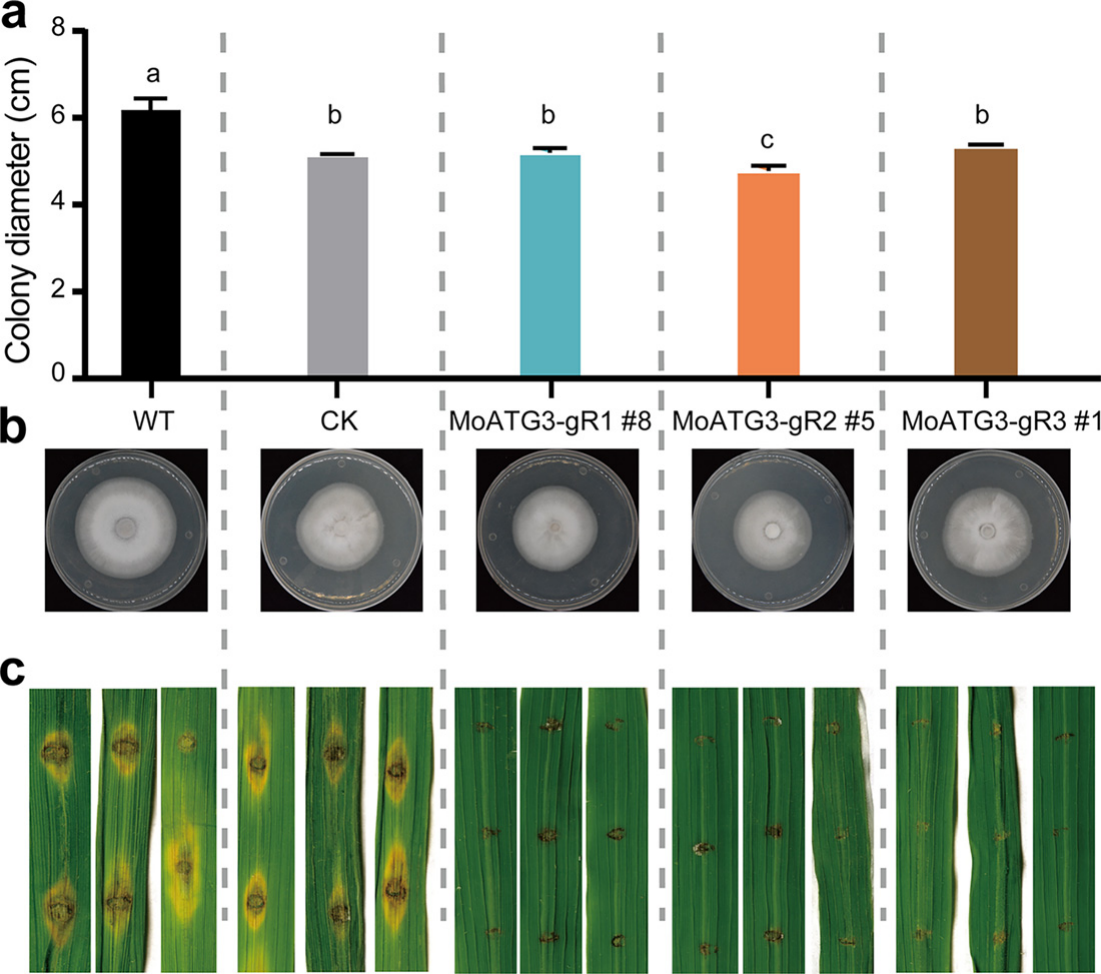
Phenotypes of *MoATG3* CRISPRi strains. (a and b) Comparisons of colony size of WT, CK, and *MoATG3* CRISPRi strains in CM plates. The photos were taken at 8 days after inoculation. Error bar, standard deviation (*n* = 4). Different letters indicate statistically significant differences determined by one-way ANOVA with *post hoc* Tukey’s HSD, α = 0.01. (c) Comparison of rice leaf blast lesions after inoculation of WT, CK, and CRISPRi strains. CK, empty vector transformants. The photos in panel c were taken at 7 days postinfection of rice leaves.

We analyzed the phenotypes of *MoATG3*-silenced strains. For each gRNA, transformants with the highest repression levels of the target gene were selected for phenotype analysis, including MoATG3-gR1-#8, MoATG3-PS2-#5 and MoATG3-PS3-#1. After 8 days of growth on complete medium (CM) plates, the diameters of colonies containing the pIF3 empty vector (CK strains) were reduced by approximately 16% compared to those of the wild-type strains (one-way analysis of variance [ANOVA] with *post hoc* Tukey’s test, *P* < 0.0001; Fig. 2a and b), suggesting that overexpression of dCas9-Mxi1 and/or selection marker genes slightly impaired M. oryzae growth. We therefore examined the phenotypes of *MoATG3* CRISPRi strains and empty vector controls. Two *MoATG3* silencing strains (MoATG3-gR1-#8 and MoATG3-gR3-#1) displayed the same growth rate as CK strains on CM plates. However, the MoATG3-gR2-#5 strain, which has the highest target gene repression, displayed a reduction in growth (Fig. 2a and b). These data imply that strong silent mutant of *MoATG3* slightly affected the growth of M. oryzae. We inoculated rice leaves with conidia from different strains. Wild-type (WT) and CK strains infected rice leaves and generated typical blast lesions with comparable sizes, suggesting that overexpression of dCas9 did not impair the pathogenesis of M. oryzae. All three *MoATG3* silencing strains only generated tiny injury lesions at the inoculation site of rice leaves rather than blast lesions, suggesting that these CRISPRi strains failed to infect rice leaves (Fig. 2c). The loss of pathogenicity of *MoATG3* CRISPRi strains is consistent with the phenotype of knockout mutants (37), indicating that CRISPRi could be used to generate knockdown mutants for gene function discovery.

### Multiplex gene silencing using arrayed tRNA-gRNA cassettes in *M. oryzae*.

**FIG 3:**
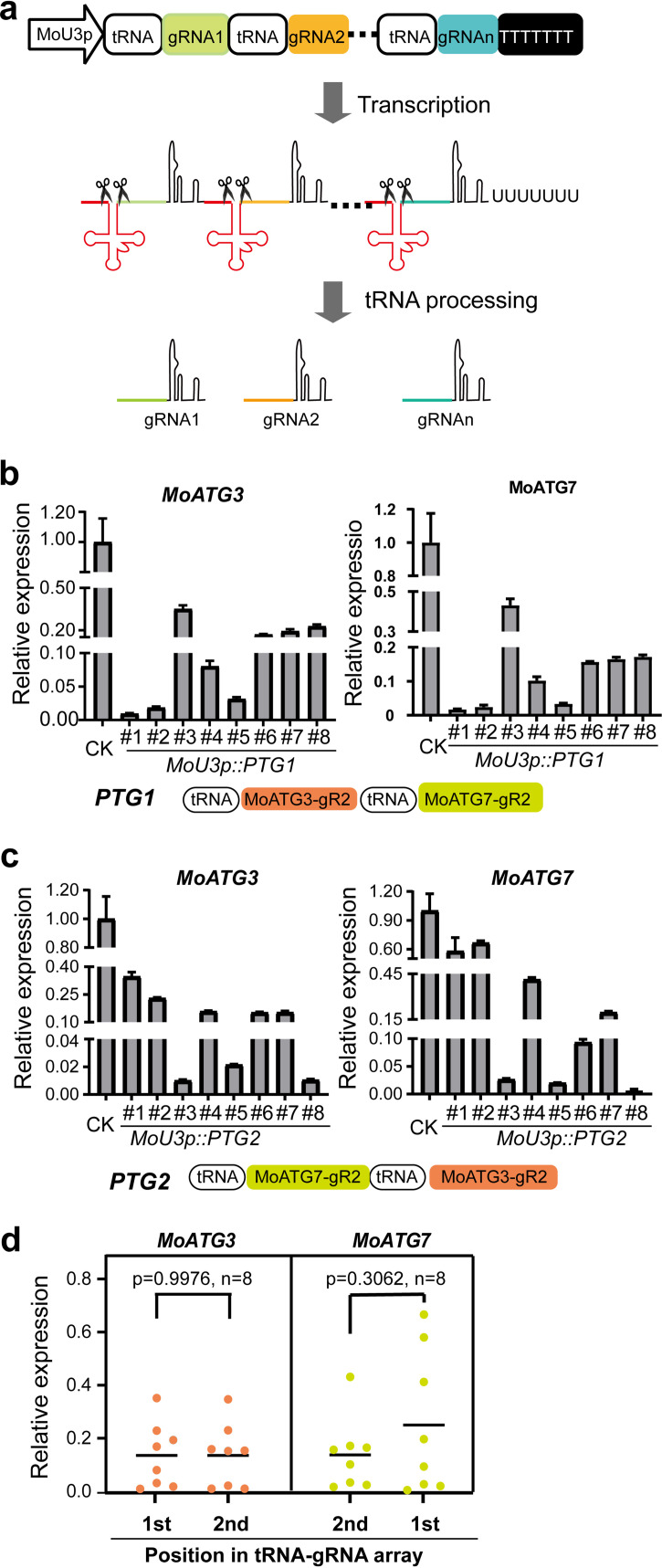
Multiplex CRISPRi in M. oryzae. (a) Schematic diagram showing the expression of multiple gRNAs using the tRNA-gRNA strategy in CRISPRi. Multiple gRNAs (gRNA1 to gRNAn) are spaced by tRNA with a poly T terminator of polymerase III. After transcription, endogenous tRNA processing RNases precisely cleave the 5′ and 3′ ends of tRNAs. As a result, all gRNAs were released to direct dCas9 to different targets. (b and c) Simultaneous silencing of *MoATG3* and *MoATG7* using two polycistronic tRNA-gRNA arrays (*PTG1* and *PTG2*) and pIF3 vector. The structures of the tRNA-gRNA arrays are shown at the bottom. CK, empty vector transformants. The plots show the relative expression of two target genes in eight transformants. Error bar, standard deviation (*n* = 3 technical replicates). (d) Comparisons of gene silencing efficiencies of gRNAs at different positions in tRNA-gRNA arrays. Each point indicates an individual transformant in panels b and c. *P* values (Student *t* test) are shown in the plot.

One attractive advantage of CRISPR/Cas9 technologies is that multiplex gene editing can be easily implemented by expressing multiple gRNAs (41). We previously showed that tandemly arrayed multiple tRNA-gRNA cassettes in one transcript can hijack the endogenous tRNA processing machinery to express different gRNAs for multiplex gene editing (Fig. 3a) (35, 42). To test the multiplex CRISPRi, we used this tRNA-gRNA strategy to simultaneously express MoATG3-gR2 and MoATG7-gR2 to generate double knockdown mutants of *MoATG3* and *MoATG7* in M. oryzae. We assembled two polycistronic tRNA-gRNA genes by switching the gRNA positions, including tRNA-[MoATG3-gR2]-tRNA-[MoATG7-gR2] (referred to as PTG1; Fig. 3b) and tRNA-[MoATG7-gR2]-tRNA-[MoATG3-gR2] (referred to as PTG2; Fig. 3c). We analyzed eight transformants for each multiplex CRISPRi construct based on the pIF3 vector, in which *MoU3* promoter is used to express polycistronic tRNA-gRNA. The RT-qPCR results showed that *MoATG3* was repressed 2- to 50-fold and that *MoATG7* was repressed 1.5- to 100-fold in these multiplex CRISPRi strains. We obtained at least three transformants that simultaneously repressed *MoATG3* and *MoATG7* >20-fold using either tRNA-gRNA architecture. The repression of two targeted genes displayed no significant differences between PTG1 and PTG2 (Student *t* test, *P* > 0.05; Fig. 3d), suggesting that the gRNA position in these two tRNA-gRNA arrays does not affect its efficiency in multiplex CRISPRi. Furthermore, the repression of target genes in multiplex CRISPRi transformants were comparable to that in single gRNA CRISPRi transformants (Fig. 1e). These observations are consistent with previous results of tRNA-based multiplex genome editing in plants and S. cerevisiae (43), indicating that multiplex CRISPRi using polycistronic tRNA-gRNA does not compromise the efficiency of each gRNA. We also tested these two tRNA-gRNA cassettes with *MoU6* promoter (pIF4 vector) and observed similar results (see Fig. S4). These data indicate that tRNA-gRNA arrays enable multiplex gene silencing in M. oryzae, which would be useful for the functional characterization of closely related genes.

10.1128/msphere.00594-22.4 FIG S4 Simultaneous silencing of *MoATG3* and *MoATG7* using tRNA-gRNA and the pIF4 vector. (a and b) Relative expression of two target genes in eight transformants. The positions of MoATG3-gR2 and MoATG7-gR2 were switched in two constructs. CK, empty vector controls. Error bars, standard deviations (*n* = 3 technical replicates). (c) Comparisons of the relative expression of *MoATG3* and *MoATG7* using different tRNA-gRNA structures. Each point indicates an individual transformant that was examined in panels a and b. Download FIG S4, JPG file, 0.7 MB. Copyright © 2023 Zhang et al. 2023 Zhang et al. https://creativecommons.org/licenses/by/4.0/ This content is distributed under the terms of the Creative Commons Attribution 4.0 International license.

### Efficient gene silencing using CRISPRi in *U. virens*.

**FIG 4:**
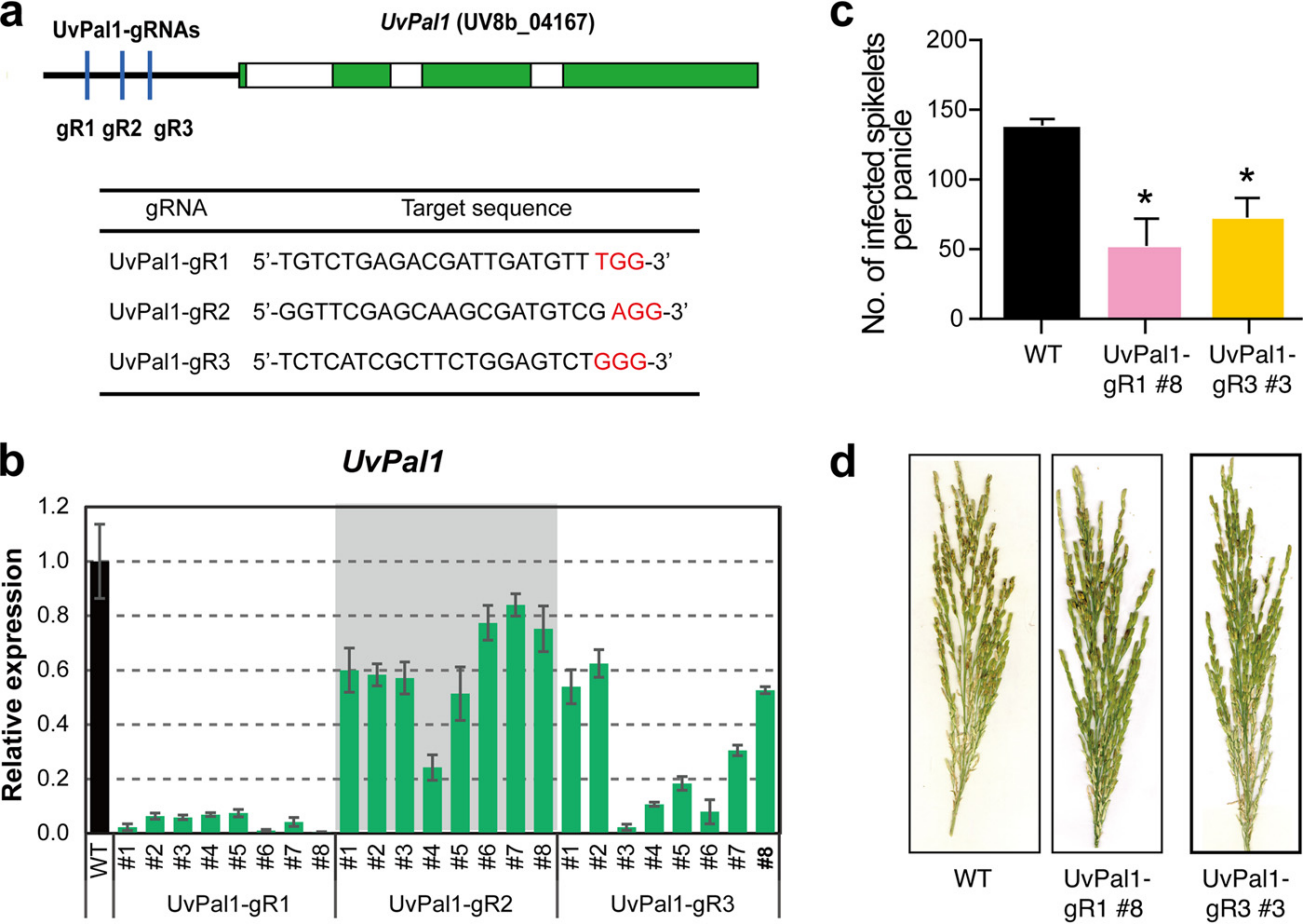
dCas9-mediated gene silencing in *U. virens*. (a) Positions and sequences of targeting sites of *UvPal1*. The upper panel shows the position of gRNAs (vertical lines with arrows), introns (white box), and protein coding regions (blue boxes). The targeting sequences of the three gRNAs are shown under the plots. Red indicates the PAM sequence for Cas9. (b) Relative expression of *UvPal1* in transformants of three gRNAs. The data are displayed as means ± the standard deviations (*n* = 3 technical replicates). (c and d) Comparison of virulence of the wild-type and CRISPRi strains. The disease symptoms were analyzed at 21 days postinoculation. The data are displayed as means ± the standard deviations.

We next sought to determine whether the pIF3 vector could efficiently silence target genes in *U. virens*. Three gRNAs were used to target the promoter of “pears and lemons” cellular morphology protein gene 1 (*UvPal1*, *UV8b_04167*) (Fig. 4a). *UvPal1* regulates hyphal growth and virulence of *U. virens* (44). The CRISPRi constructs and empty vectors were transformed into *U. virens* isolate HWD-2 by ATMT. Although dCas9-Mxi1 and gRNA were expressed using promoters from M. oryzae, the pIF3 vector displayed efficient target gene repression in *U. virens* as in M. oryzae. Compared to WT strains, *UvPal1* was repressed to variable levels in CRISPRi transformants (Fig. 4b). UvPal1-gR1 suppressed target genes 12- to 100-fold, which was higher than the other two gRNAs (Fig. 4b). We tested the virulence of two CRISPRi strains (UvPal1-gR1-#8 and UvPal1-gR3-#3) that show strong transcriptional suppression of *UvPal1*. Although the phenotypes of *UvPal1* CRISPRi strains were weaker than those of *UvPal1* knockout strains, which completely lost virulence to infect rice flowers (44), *UvPal1* CRISPRi strains displayed reduced virulence. As shown in Fig. 4c and d, two *UvPal1* CRISPRi strains produced fewer smut balls than WT strains (Student *t* test, *P* < 0.01). Together, these data suggest that CRISPRi is highly efficient in *U. virens* and would be a useful tool for fast discovery of gene function in pathogenic fungal species.

## DISCUSSION

In this study, we present an efficient and robust CRISPRi toolkit for targeted gene silencing in two filamentous fungal pathogens, M. oryzae and *U. virens*. We used nine gRNAs to target the promoters of three genes and demonstrated that the optimized pIF series of vectors repressed target gene expression >100-fold. More importantly, multiple genes could be simultaneously silenced using the tRNA-gRNA strategy (Fig. 3), which would be particularly useful to study closely related genes, fine-tuning the regulatory pathways, and repurposing metabolic pathways in filamentous fungi. In the future, a large population of gRNAs could be synthesized to generate a CRISPRi library to perturb gene expression for fast gene function discovery in fungal pathogens.

Several reports indicate that Cas9 is toxic to some fungal species, such as Schizosaccharomyces pombe (45), S. cerevisiae (46), Cryptococcus neoformans (47), and M. oryzae (25). Therefore, targeted gene knockout based on NHEJ repair is not feasible, but HR-based gene replacement using transient expression of Cas9 was used in these fungal species. The nonspecific cytotoxicity of Cas9-cleavage is likely due to DNA damage and the subsequent repair response (48, 49). Indeed, a survey of DSB repair preference in four yeast species found that S. cerevisiae has much higher HDR than Y. lipolytica, K. marxianus, and *S. stipitis* (50). A recent study analyzed Cas12a-mediated gene replacement in M. oryzae and found significant variation in DNA repair profiles, ranging from small indels to kilobase size deletions and insertions (51), providing important clues of Cas cytotoxicity in some fungal species. CRISPRi uses dCas9, which has no toxicity in M. oryzae and *U. virens*, providing a simple genetic tool to manipulate gene expression. Despite the slight reduction in growth in M. oryzae, which is likely due to the fitness cost of overexpressing foreign proteins, M. oryzae and *U. virens* strains containing empty CRISPRi vectors displayed normal development and pathogenicity as WT strains (Fig. 2). We anticipated that CRISPRi could be used to interrogate gene function by comparing phenotypes of CRISPRi strains with empty vector transformants or WT strains. Indeed, silencing *MoATG3* completely abolished the pathogenicity of M. oryzae (Fig. 2), and silencing *UvPal1* significantly reduced the virulence of *U. virens* (Fig. 4). Both examples indicate that CRISPRi would be a powerful tool to discover genes that regulate development, growth, infection, and pathogenicity of fungal pathogens.

We optimized different components of the CRISPRi toolkit. For transcriptional repression domains, Mxi1 has slightly higher efficiency than 3× SRDX in M. oryzae (Fig. 1d). Both the *MoU3* and *MoU6* promoters efficiently expressed gRNAs (Fig. 1e and Fig. 4; see also Fig. S4). However, the choice of promoter to express dCas9-Mxi1 has a considerable effect on CRISPRi efficiency. Compared to *MoTEF1* promoter, expressing dCas9-Mxi1 with *MoRP27* promoter significantly enhanced CRISPRi efficiencies in M. oryzae. Furthermore, a single gRNA is sufficient to silence the target gene, but the distance between the targeting site and TSS affects repression efficiency. Among the different gRNAs we tested, the highest silencing efficiencies were observed in gRNAs whose targeting sites were located 100 to 200 bp upstream of transcription start sites in M. oryzae. Similar results were observed in CRISPRi in S. cerevisiae (52) and in CRISPR activation in human cells (14). The optimal distance between the targeting site and TSS might be affected by the transcriptional repressor domain of the CRISPRi vector and potentially by the chromatin opening status. We also introduced the tRNA-gRNA strategy to simultaneously silence two genes in M. oryzae (Fig. 3; see also Fig. S4). This strategy was previously used in CRISPR/Cas9 genome editing in many eukaryotic organisms. For example, a total of 2 to 8 targets were simultaneously edited using one polycistronic tRNA-gRNA transcript in rice (35), human cells (53, 54), and S. cerevisiae (43). Those studies demonstrated that the editing efficiencies of each gRNA were not affected by its position in a tRNA-gRNA array (35, 43). Similarly, this study found that gRNA positions in bicistronic tRNA-gRNA transcripts did not affect CRISPRi efficiencies in M. oryzae (Fig. 3; see also Fig. S4). In addition to the tRNA-gRNA strategy, the Csy4-based processing of polycistronic gRNAs (55) and Cas12a (56) systems were also used in CRISPRi in S. cerevisiae. It will be interesting to compare the target number and efficiencies of different multiplex strategies for CRISPRi in fungi in the future.

Although CRISPRi efficiently suppressed gene transcription in M. oryzae and *U. virens*, the potential limitations of this tool should be considered for gene function studies in plant fungal pathogens. First, highly specific gRNA should be used to eliminate or minimize the off-target effect of Cas9. Because Cas9 tolerates one or two mismatches at the PAM-distant region, dCas9 may recognize and silence unintended genes if low specific gRNAs were used in CRISPRi. The on- and off-targeting rules of Cas9 in genome editing have been extensively studied (57), and many bioinformatic tools (58) could be used to design target-specific gRNAs when the genome sequence is available. In addition, high-fidelity Cas9 variants (58) could be used to alleviate the off-target risk of low specific gRNAs in CRISPRi. Second, the CRISPRi fungal strains developed in this study carry T-DNA insertions in their genomes. Although T-DNA tends to integrate at the intergenic regions (59), the possibility that T-DNA insertion may disrupt the expression of unintended target genes cannot be ignored in gene function studies. Therefore, several individual transformants with different T-DNA insertion events should be used as biological replicates in all experiments. The T-DNA insertion site may also affect the expression of dCas9-Mxi1 and gRNAs, which may explain the variation of target gene repression levels of different individual transformants. Third, the PAM constraints of Cas9 might restrict the availability of gRNAs with high CRISPRi efficiencies. Cas9 from Streptococcus pyogenes recognizes G-rich PAM, while the promoter regions often contain AT-rich sequences. To expand the targeting space, PAM-relaxed Cas9 variant and Cas12 (56), which recognize alternative PAM sequences, could be used in CRISPRi tools for fungal pathogens.

Together, this study presents a CRISPRi toolkit for targeted gene silencing in M. oryzae and *U. virens*. Given its simplicity, robustness, and high efficiency, CRISPRi would facilitate gene function discovery in fungal pathogens and potentially enable high-throughput genetic screening in the future.

## MATERIALS AND METHODS

### Fungal strains, culture conditions, and plant materials.

M. oryzae isolate 70-15 and *U. virens* strain HWD-2 were used in this study. Agrobacterium tumefaciens strain EHA105 was used for fungal transformation. WT and CRISPRi M. oryzae strains were cultured on complete medium (CM) containing 6 g/L yeast extract, 3 g/L enzymatic casein hydrolysate, 3 g/L acidic casein hydrolysate, 10 g/L glucose, and 15 g/L agar. For rice inoculation, M. oryzae strains were cultured on oatmeal tomato agar medium (40 g/L boiled oatmeal filtrate, 150 mL tomato juice, and 20 g/L agar) at 28°C. *U. virens* strains and transformants were cultured on potato sucrose broth (PSB) medium or potato sucrose agar plates at 28°C.

### Construction of pIF vectors.

The DNA assembly procedure of the pIF1 to pIF4 vectors is shown in Fig. S5 and S6. Briefly, the DNA parts used to assemble CRISPRi vectors were cloned from the following templates. The vector backbone is p1300-BsaI, which was derived from pCAMBIA1300 by removing all BsaI sites (35). The *dCas9* was cloned from pAC149-pCR8-dCas9VP160, which was a gift from Rudolf Jaenisch (Addgene plasmid 48221) (60); the codon-optimized 3×SRDX sequence was synthesized from GenScript Biotech (see its sequence in Fig. S1); *Mxi1* was cloned from the pTDH3-dCas9-Mxi1 plasmid, which was a gift from Stanley Qi and Jonathan Weissman (Addgene plasmid 46921) (13); the *MoTEF1* promoter was amplified from the M. oryzae isolate 70-15 genome; and the *MoRP27* promoter was amplified from the pKN plasmid from Xiao-Lin Chen at Huazhong Agricultural University. Before assembly of these vectors, the BsaI site in the *MoRP27* promoter was removed using a site-directed mutagenesis kit (TaKaRa). Figures S5 and S6 show the procedures and cloning methods used to assemble these DNA parts into CRISPRi vectors. See Table S1 for the primers used for vector assembly in this study. The pIF vectors described in this study are deposited in Addgene (plasmid IDs 196066 to 196069).

10.1128/msphere.00594-22.5 FIG S5 Flow chart showing the DNA assembly steps used to construct pIF1 and pIF2. Download FIG S5, JPG file, 1.4 MB. Copyright © 2023 Zhang et al. 2023 Zhang et al. https://creativecommons.org/licenses/by/4.0/ This content is distributed under the terms of the Creative Commons Attribution 4.0 International license.

10.1128/msphere.00594-22.6 FIG S6 Flow chart showing the DNA assembly steps used to construct pIF3 and pIF4. Download FIG S6, JPG file, 1.3 MB. Copyright © 2023 Zhang et al. 2023 Zhang et al. https://creativecommons.org/licenses/by/4.0/ This content is distributed under the terms of the Creative Commons Attribution 4.0 International license.

10.1128/msphere.00594-22.7 TABLE S1 Primers used in this study. Download Table S1, DOCX file, 0.02 MB. Copyright © 2023 Zhang et al. 2023 Zhang et al. https://creativecommons.org/licenses/by/4.0/ This content is distributed under the terms of the Creative Commons Attribution 4.0 International license.

The target-specific gRNAs were designed using a bioinformatic pipeline described in CRISPR P 2.0 (61). *MoATG3* and *MoATG7* information were obtained from M. oryzae genome annotation (accession number of assembly GCA_000002495.2). The *UvPal1* gene information was downloaded from *U. virens* genome annotation (accession number GCA_000687475). Of note, transcription start site of *UvPal1* was undetermined yet. For gRNA cloning, tRNA and gRNA were first fused using GoldenGate cloning (New England Biolabs) and then inserted into the BsaI sites of pIF vectors as described previously (35). See Table S1 for primers used for gRNA cloning in this study.

### *Agrobacterium*-mediated transformation of *M. oryzae* and *U. virens*.

*Agrobacterium*-mediated transformation was used to deliver CRISPRi constructs into M. oryzae and *U. virens*. M. oryzae transformation was performed as described by Chen et al. (9). The *U. virens* transformation was performed as described previously (10, 11). After two rounds of selection of positive transformants using 200 μg/mL of hygromycin, individual positive colonies were confirmed using vector specific primers and stored in filter paper at –20°C for further analysis.

### Total RNA extraction and quantitative RT-PCR.

For M. oryzae, conidia were cultured in 50 mL of liquid CM media at 28°C for 4 days. For *U. virens* RNA extraction, transformants were cultured in 50 mL of PSB medium at 28°C for 7 days. After harvesting the mycelium, the total RNA was extracted using TRIzol reagent (Thermo-Fisher Scientific). For reverse transcription, 1.5 μg of total RNA was treated with DNase I (1.25 U; New England BioLabs) to remove genomic DNA contamination. Then, the RNA samples were incubated at 70°C for 10 min to inactivate DNase I. Reverse transcription was performed using MMLV reverse transcriptase according to the manufacturer’s instructions (TaKaRa). Real-time PCR was performed using QuantStudio 3 (Thermo Fisher Scientific) and TB Green Premix *Ex Taq* II (TaKaRa). The relative expression of the target gene was calculated using the 2^–ΔΔ^*^CT^* method (62) using elongation factor 1 and β-tubulin as internal reference genes for M. oryzae and *U. virens*, respectively. The primers for RT-qPCR are listed in Table S1.

### Plant material and inoculation assay.

For M. oryzae inoculation, rice (Oryza sativa) cv. CO-39 was used in this study. The preparation of M. oryzae conidia and leaf inoculation were performed as described previously (21). For *U. virens* inoculation, rice (Oryza sativa) cv. Wanxian-98 was inoculated as described by Chen et al. (44). *U. virens* conidia were adjusted to 10^6^ spores/mL and then injected into the middle of young panicles using a syringe. Inoculated plants were grown in a greenhouse (humidity, 95%; temperature, 25°C) for 21 days.
