# Supplementary figures and images for: CRISPR/dCas9-Mediated Gene Silencing in Two Plant Fungal Pathogens
Source: mSphere. 2023 Jan 19;8(1):e00594-22. doi: 10.1128/msphere.00594-22 (PMC9942560; doi:10.1128/msphere.00594-22)

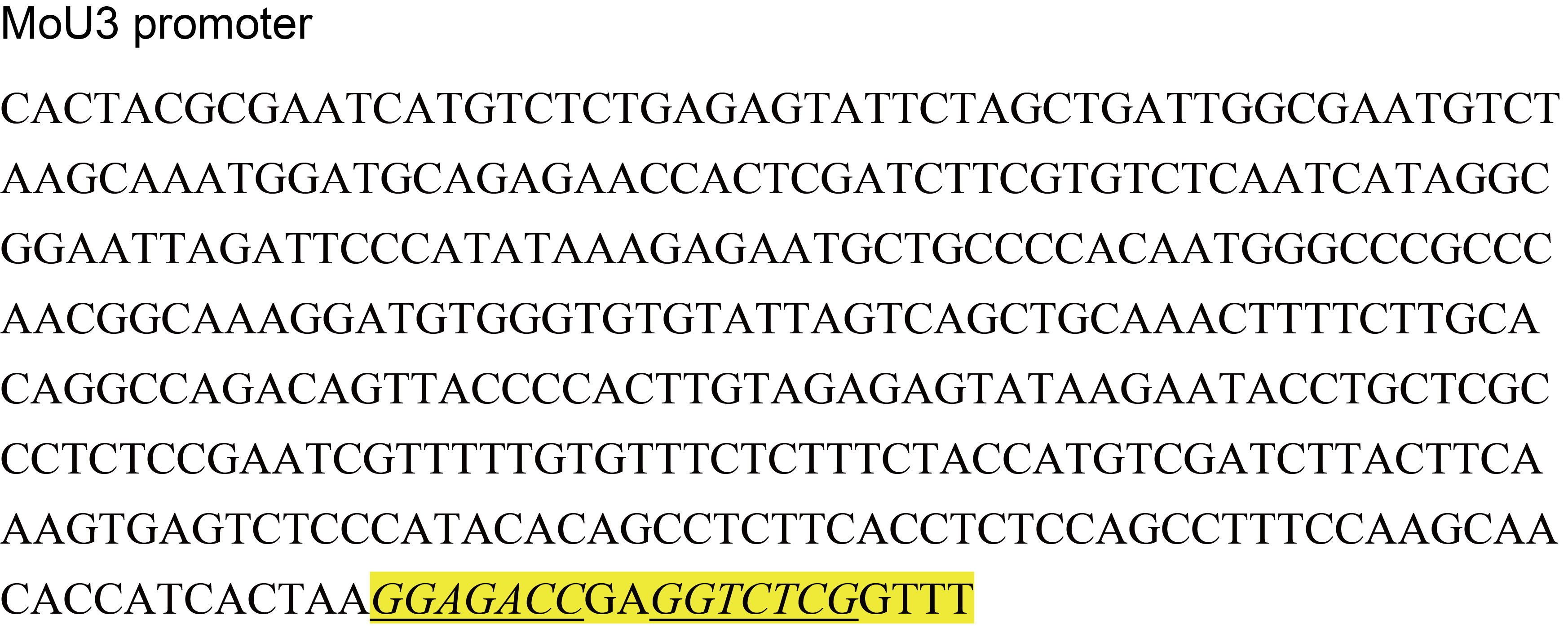

Supplement: FIG S2 [file msphere.00594-22-s0002.jpg]

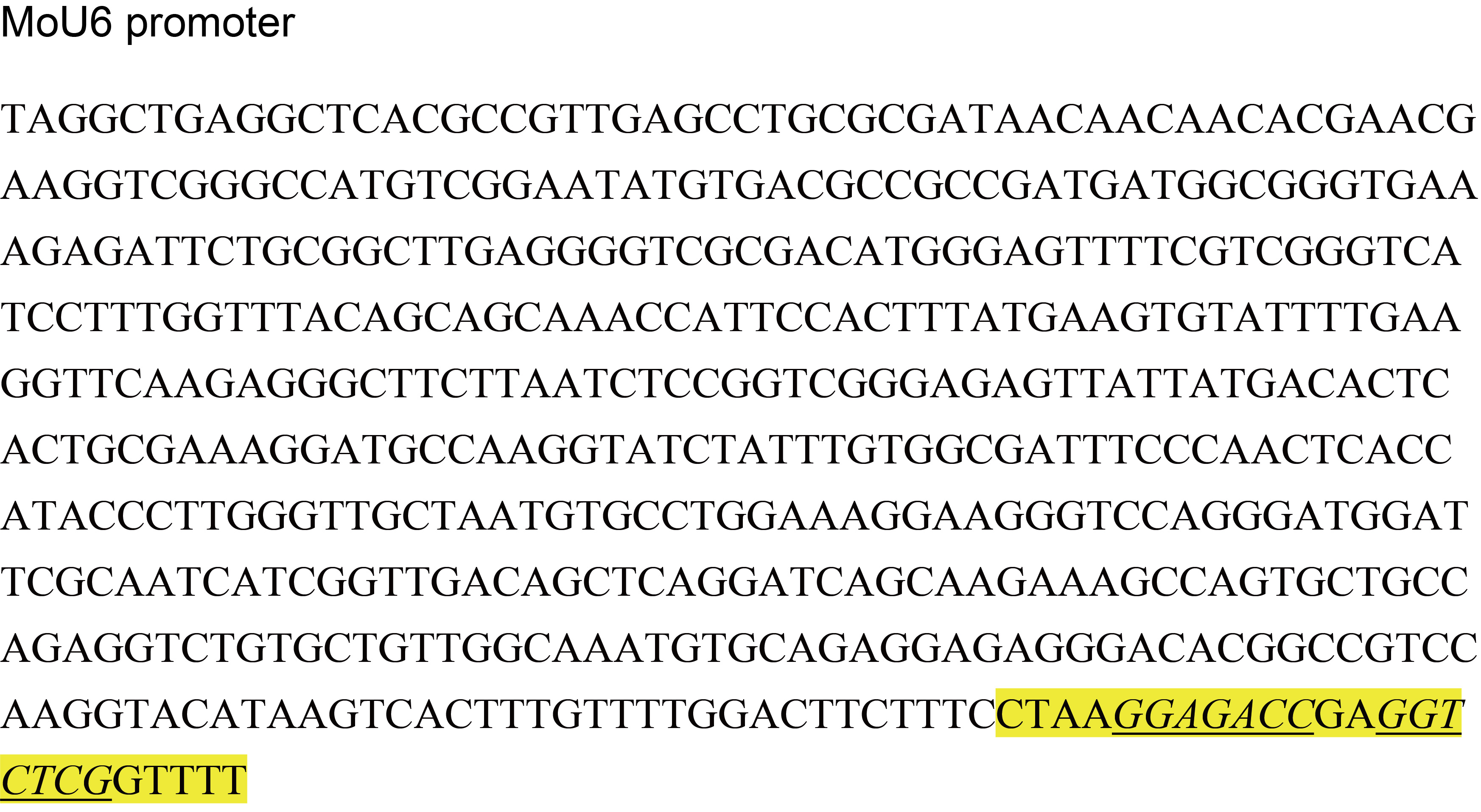

Supplement: FIG S3 [file msphere.00594-22-s0003.jpg]

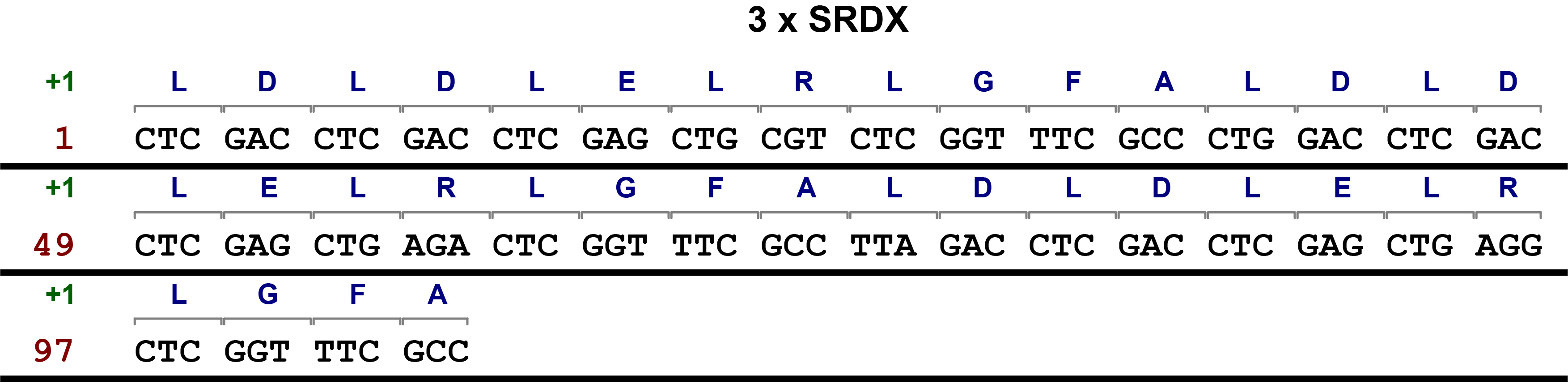

Supplement: FIG S1 [file msphere.00594-22-s0001.jpg]

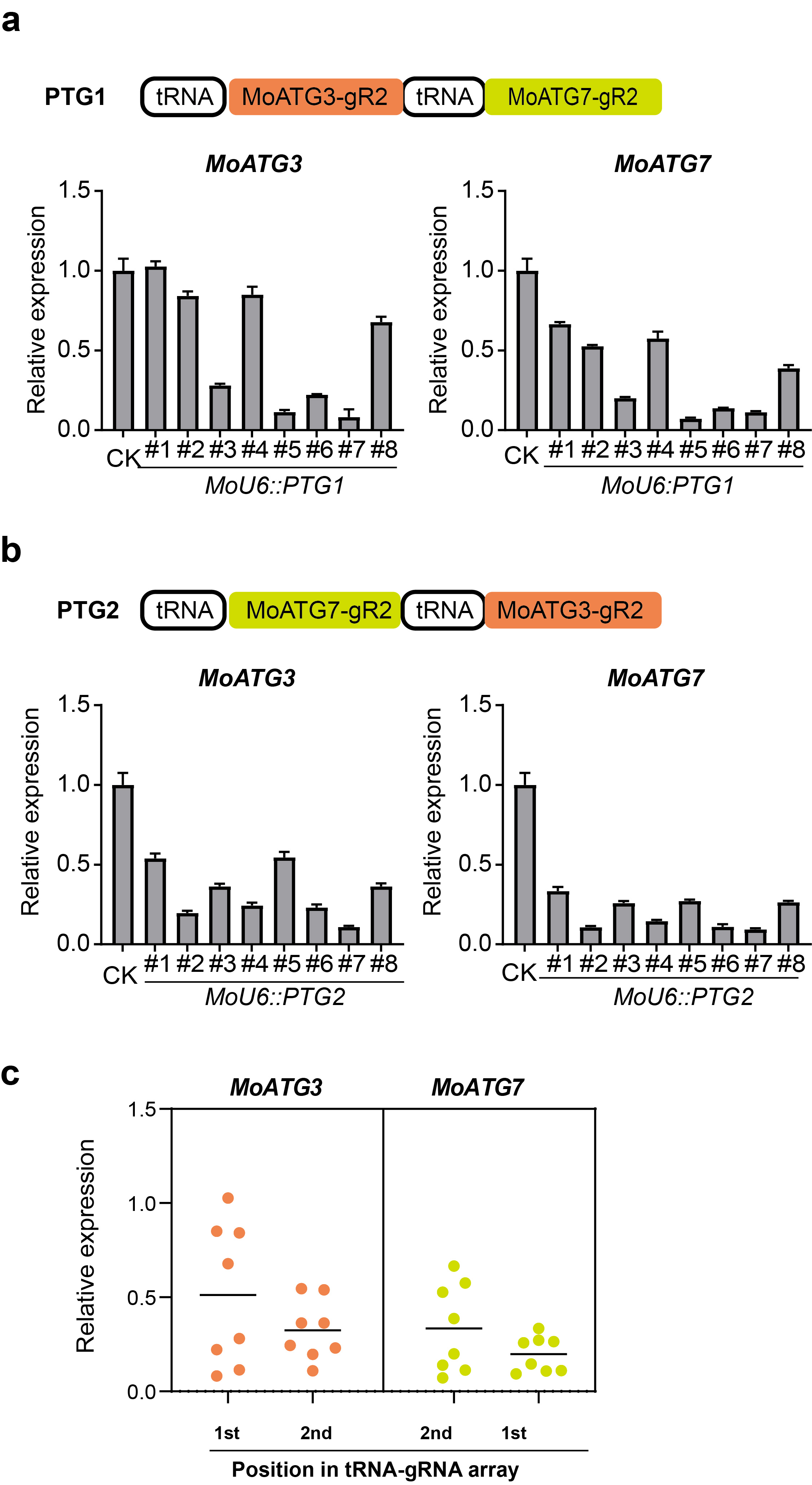

Supplement: FIG S4 [file msphere.00594-22-s0004.jpg]

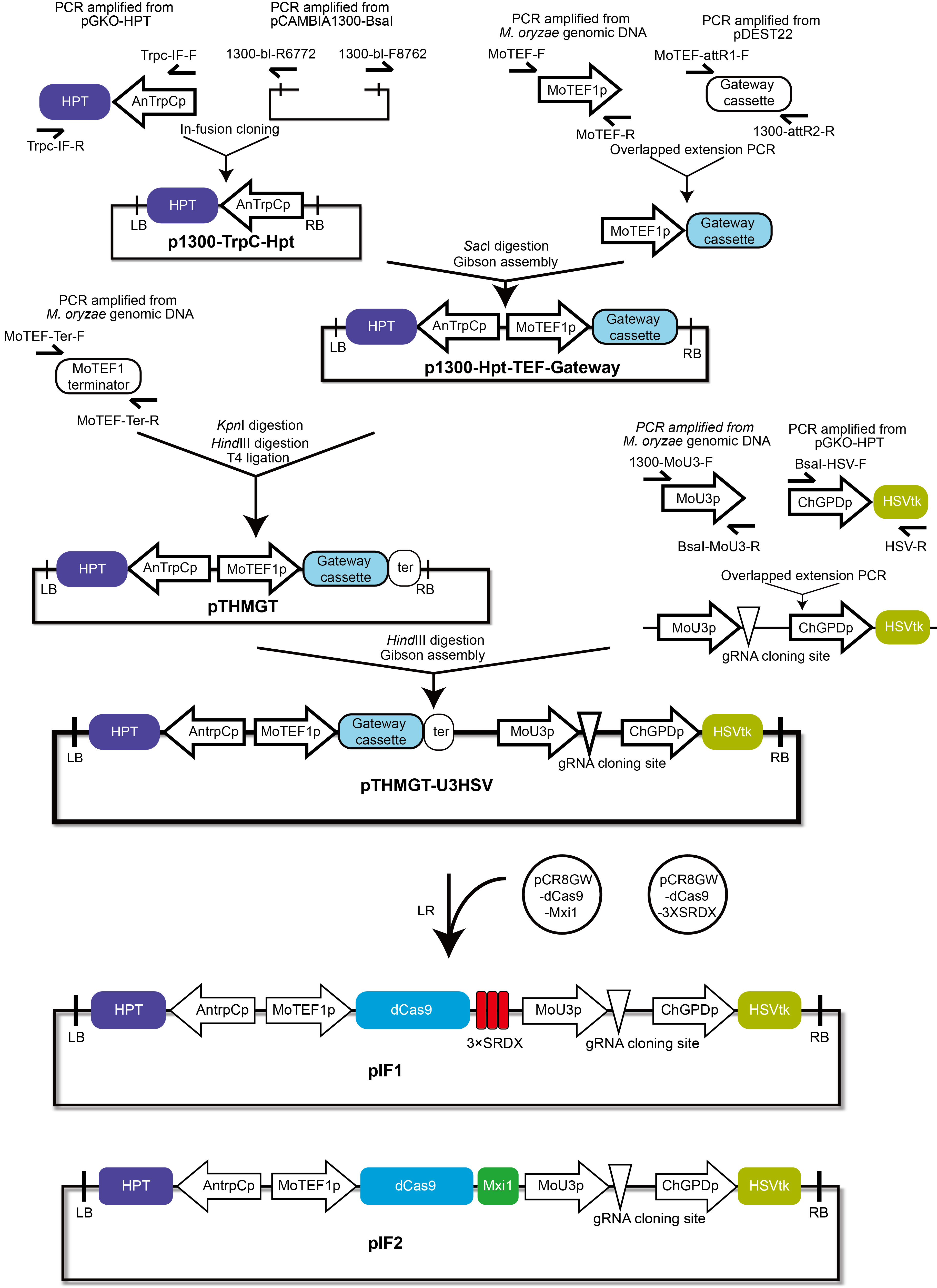

Supplement: FIG S5 [file msphere.00594-22-s0005.jpg]

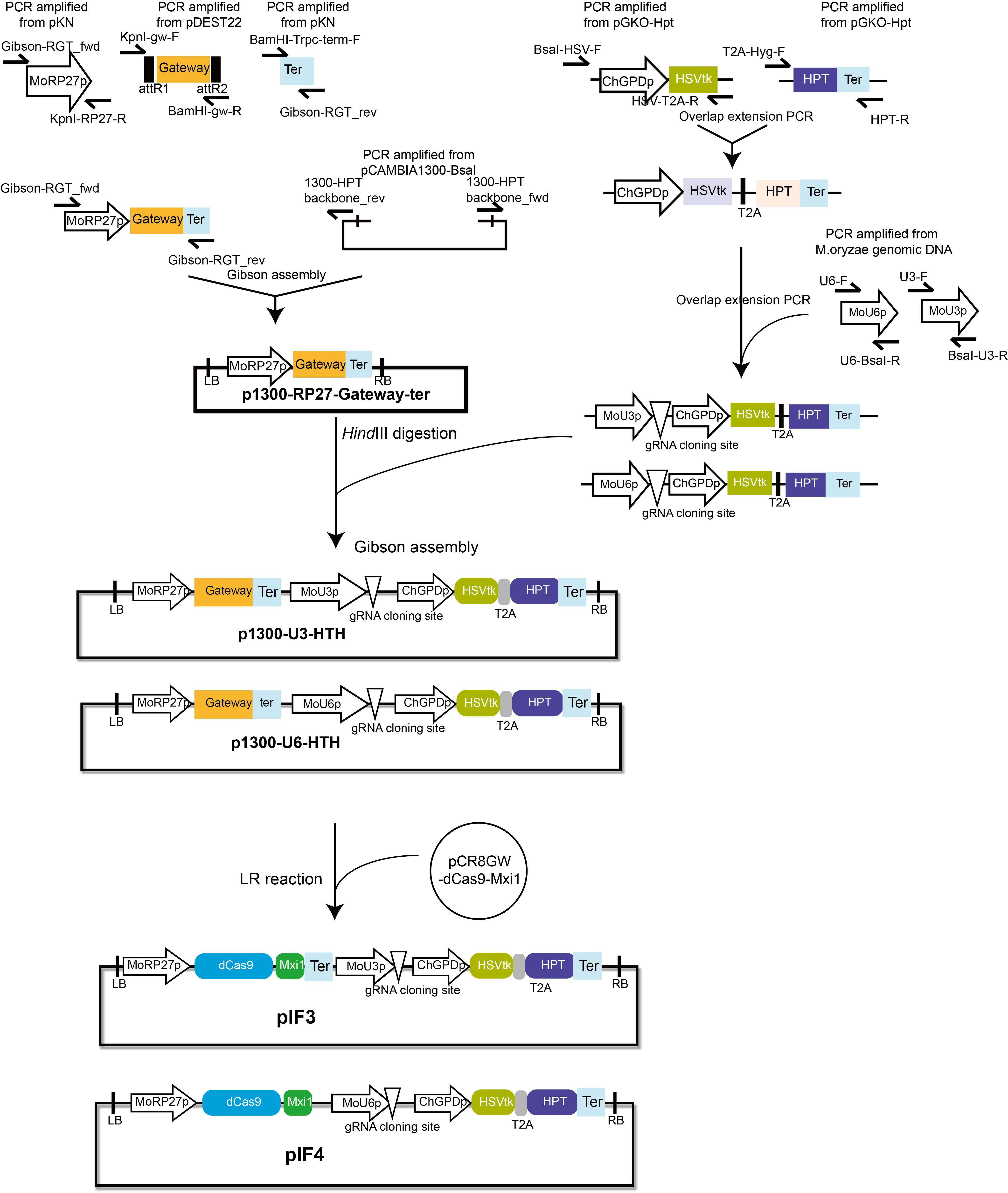

Supplement: FIG S6 [file msphere.00594-22-s0006.jpg]
