# Supplementary material for: CRISPR/dCas9-Mediated Gene Silencing in Two Plant Fungal Pathogens
Source: mSphere. 2023 Jan 19;8(1):e00594-22. doi: 10.1128/msphere.00594-22 (PMC9942560; doi:10.1128/msphere.00594-22)
Supplement: TABLE S1 [file msphere.00594-22-s0007.docx]

**Table S1** **Primers used in this study**

| **Primer name** | **Sequence (5’-3’)** | **Purpose** |
| --- | --- | --- |
| **Primers used for CRISPRi vector construct** | | |
| M-pKN-BsaI | AGCAGATCAACGGTCGTCAATGGACCTACGAGACTGAG | Mutagenesis of *Bsa*I recognition site |
| M-pD22-BamHI | CGGGGCGTAATCTAGAGCATCCGGCTTACTAAAAG | Mutagenesis of *Bam*HI recognition site |
| Gibson-RGT_fwd | ATATGCTCAACACATGAGCGGAATTCATAAATGTAGGTATTACC | *MoRP27* promoter cloning |
| KpnI-RP27-R | GGTACCTTTGAAGATTGGGTTCCTA |  |
| KpnI-gw-F | GGTACCACAAGTTTGTACAAAAAAGC | Gateway cassette cloning (attL1-attL2) |
| BamHI-gw-R | GGATCCACCACTTTGTACAAGAAAG |  |
| BamHI-Trpc-term-F | CTTGTACAAAGTGGTGGATCCACTTAACGTTACTG | *AnTrpC* terminator cloning |
| Gibson-RGT_rev | TGCATTAATGAATCGGCCAAAAGCTTAAAGAAGGATTACC |  |
| 1300-HPT backbone_fwd | AATCCTTCTTTTGGCCGATTCATTAATGC | pCAMBIA1300-HPT vector backbone amplification |
| 1300-HPT backbone_rev | TTATGGTACCCGCTCATGTGTTGAGCATATAAG |  |
| BsaI-HSV-F | GGAGACCGAGGTCTCGGTTTTGAATTGGGTACTCAAATTGGTTC | HSVtk-T2A amplification |
| HSV-T2A-R | ACGACCCTCGGCACGGTTAGCCTCCCCCATCT |  |
| T2A-HPT-F | CTCCTCACCTGTGGTGACGTCGAGGAGAACCCTGGTCCTATGAAAAAGCCTGAACTCAC | T2A-HPT-AnTrpC ter amplification |
| HPT-R | TAATGCCTCAGCACTAGTCGGGGGA |  |
| U6-F | atgcaTAGGCTGAGGCTCACGCCGTTG | *MoU6* promoter cloning |
| M-U6-BsaI-R | ATGATTGCGAatCCATCCCTGGACCCTTCCTTTCCAGGC |  |
| M-U6-BsaI-F | AGGGATGGatTCGCAATCATCGGTTGACAGCTCAG |  |
| U6-BsaI-R | AAAACCGAGACCTCGGTCTCCGAAAGAAGTCCAAAACAA |  |
| U3-F | CACTACGCGAATCATGTCTCTGAGAGT | *MoU3* promoter cloning |
| BsaI-MoU3-R | CGAGACCTCGGTCTCCTTAGTGATGGTGTTGCTTGGAAA |  |
| gibson-U3p-F | AGTCGACCTGCAGGCATGCACACTACGCGAATCATGTC | Gibson assembly primer for MoU3-HSV-HPT amplification |
| Gibson-U6-F | AGTCGACCTGCAGGCATGCATAGGCTGAGGCTCACGCC | Gibson assembly primer for MoU6-HSV-HPT amplification |
| Gibson-Hyg-R | TAAAACGACGGCCAGTGCCATAATGCCTCAGCACTAGTC | General reverse primer for Gibson assembly of MoU3-HSV-HPT or MoU3-HSV-HPT |
| Gibson-SRDX-F | GAGGCCAGCGGGCCGGCCGGATCCCTCGACCT | 3×SRDX cloning |
| Gibson-SRDX-R | ATTCTTAATTAATCAATCGATTCAGGCGAA |  |
| BamHI-Mxi1-F | ACGGATCCATGGAACGTGTGAGAAT | Mxi1 cloning |
| ClaI-Mxi1-R | TCATCGATTTAGCCTCTGGGAGAGGG |  |
| **Primers used for gRNA cloning using Golden Gate assembly** | | |
| L8AD5-F | CGGGTCTCAGGCAGGATGGGCAGTCTGCTAAACAAAGCACCAGTGG | General PCR primer to amplify DNA parts of tRNA-gRNA for CRISPRi vector |
| L3AD5-R | TAGGTCTCCAAACGGATGAGCGACAGCAAACAAAAAAAAAAGCACCGACTCG |  |
| S8AD5-F | CGGGTCTCAGGCAGGATGGGCAGTCTGCTAA | General PCR primer to amplify assembled tRNA-gRNA fragment for CRISPRi vector |
| L3AD6-R | TAGGTCTCCAAACGGATGAGCGACAGCAAACAAAAAAAAAATGCACCAGCCGGG |  |
| INMoATG3-PS1-F | TAGGTCTCC TATGTCTCTGTCTAGTTTTAGAGCTAG | Target specific primer to amplify MoATG3-gR1 to assemble tRNA-gRNA |
| INMoATG3-PS1-R | CGGGTCTCA CATACAAGTCTGCACCAGCCGGG |  |
| INMoATG3-PS2-F | TAGGTCTCCCGATCTGATATTCAGTTTTAGAGCTAG | Target specific primer to amplify MoATG3-gR2 to assemble tRNA-gRNA |
| INMoATG3-PS2-R | CGGGTCTCA ATCGGGTACATGCACCAGCCGGG |  |
| INMoATG3-PS3-F | TAGGTCTCCGCTGATAACGCCCCGTTTTAGAGCTAG | Target specific primer to amplify MoATG3-gR3 to assemble tRNA-gRNA |
| INMoATG3-PS3-R | CGGGTCTCACAGCCTTATCTGCACCAGCCGGG |  |
| INMoATG7-PS1-F | TAGGTCTCCAACAGAGCATGACCGTTTTAGAGCTAG | Target specific primer to amplify MoATG7-gR1 to assemble tRNA-gRNA |
| INMoATG7-PS1-R | CGGGTCTCATGTTGGCGGGTGCACCAGCCGGG |  |
| INMoATG7-PS2-F | TAGGTCTCCATGCCACATGCCCAGTTTTAGAGCTAG | Target specific primer to amplify MoATG7-gR2 to assemble tRNA-gRNA |
| INMoATG7-PS2-R | CGGGTCTCAGCATTTTTAGTGCACCAGCCGGG |  |
| INMoATG7-PS3-F | TAGGTCTCCTTATCTAATGCATTGTTTTAGAGCTAG | Target specific primer to amplify MoATG7-gR3 to assemble tRNA-gRNA |
| **Primers used for RT-qPCR in *M. oryzae*** | | |
| RT-MoATG3-F | TTGACAGCTGGTCCATTCCC | *MoATG3* RT-qPCR primer |
| RT-MoATG3-R | TCTTGTTGCGCCAAGAAAGC |  |
| RT-MoATG7-F | TAGGTGAGCGGTGTTAGTGC | *MoATG7* RT-qPCR primer |
| RT-MoATG7-R | CAGTCGATCACCACACTCCC |  |
| RT-MoEf1-F | CATCTTAACGTCGTCGTCATC | *MoEf1* RT-qPCR primer |
| RT-MoEf1-R | AGTGGCCGGTAGTCGTGG |  |
| **Primers used for gRNA cloning for *U. virens*** | | |
| S5-MoS-F | GCATCAGGATGAGCGACAGCTGCA | General forward primer for gRNA cloning in pIFU3-U3 for *U.virens* |
| S3-MoS-R | AAGCGTGGATGGGCAGTCTGAAAC |  |
| UvPal1-PS1 | AGCGACAGCTGCATGTCTGAGACGATTGATGTTGTTTCAGACTGCC | UvPal1-gR1 oligo |
| UvPal1-PS2 | AGCGACAGCTGCAGTTCGAGCAAGCGATGTCGGTTTCAGACTGCC | UvPal1-gR2 oligo |
| UvPal1-PS3 | AGCGACAGCTGCATCTCATCGCTTCTGGAGTCTGTTTCAGACTGCC | UvPal1-gR3 oligo |
| **Primers used for RT-qPCR for *U. virens*** | | |
| RT-UvPal1-F | GGTTCACGGCGACGAGAGT | *UvPal1* RT-qPCR primer |
| RT-UvPal1-R | GCAGCCGGTGTTCCTTCA |  |
| RT-Uv-β-tublin-F | GGGGCCACATGACAAGAGAA | *Uv-β-tublin* RT-qPCR primer |
| RT-Uv-β-tublin-R | ACGTCGAGGTTCAGTGTTCC |  |
